# Transcriptome Classification Reveals Molecular Subgroups in Idiopathic Pulmonary Fibrosis

**DOI:** 10.1155/2022/7448481

**Published:** 2022-07-16

**Authors:** Yuxia Liu, Chang Xu, Wenxin Gao, Huaqiong Liu, Chenglong Li, Mingwei Chen

**Affiliations:** ^1^Department of Respiratory and Critical Care Medicine, The First Affiliated Hospital of Xi'an Jiaotong University, Xi'an, Shanxi, China; ^2^Library of Binzhou Medical University, Yantai, Shandong, China; ^3^Department of Radiology, Binzhou Medical University Hospital, Binzhou, Shandong, China; ^4^Department of Neurosurgery, Binzhou Medical University Hospital, Binzhou, Shandong, China

## Abstract

Idiopathic pulmonary fibrosis (IPF) is a disease of progressive lung fibrosis with a high mortality rate. This study aimed to uncover the underlying molecular features for different types of IPF. IPF microarray datasets were retrieved from GEO databases. Weighted gene co-expression analysis (WGCNA) was used and identified subgroup-specific WGCNA modules. Infiltration-level immune cells in different subgroups of microenvironments were analyzed with CIBERSORT algorithms. The result is we classified 173 IPF cases into two subgroups based on gene expression profiles, which were retrieved from the GEO databases. The SGRQ score and age were significantly higher in C2 than in C1. Using WGCNA, five subgroup-specific modules were identified. M4 was mainly enriched by MAPK signaling, which was mainly expressed in C2; M1, M2, and M3 were mainly enriched by metabolic pathways and Chemokine signaling, and the pathway of M5 was phagosome inflammation; M1, M2, M3, and M5 were mainly expressed in C1. Utilizing the CIBERSORT, we showed that the number of M1 macrophage cells, CD8 T cells, regulatory T cells (Tregs), and Plasma cells was significantly different between C1 and C2. We found the molecular subgroups of IPF revealed that cases from different subgroups may have their unique patterns and provide novel information to understand the mechanisms of IPF itself.

## 1. Introduction

Idiopathic pulmonary fibrosis is a chronic progressive disease characterized by excessive wound repair and interstitial pneumonia [1, 2]. It is one of the most common refractory diseases of the respiratory system that poses a serious threat to the life and health of human beings. The worldwide incidence of IPF is about (2∼29)/100,000 and has been increasing annually, while the median survival period is 3∼5 years after diagnosis [3, 4]. Idiopathic pulmonary fibrosis may be driven by abnormal epithelium and transformed by heterogeneous fibroblasts in different activation states [5]. The 5-year survival rate of patients with late-stage IPF is much lower than that of early-stage IPF [6]. Although new evidence suggests that there is a genetic association between early and late disease, the differences in gene expression among different stages of IPF have not been investigated. Therefore, it is still difficult to fully understand the mechanisms underlying the pathogenesis and progression of IPF.

Microarrays are a recent high-throughput technology used for gene expression analysis [7]. High-throughput techniques are increasingly being used to identify the molecular mechanisms underlying the pathogenesis or progression of IPF. However, most studies have focused on the differences between IPF cases and normal controls, neglecting the differences among different stages of IPF. In this study, we used bioinformatics analysis to analyze the gene expression data of 173 IPF patients and explored the differences among different IPF subtypes.

## 2. Materials and Methods

### 2.1. Microarray Data Information

Microarray datasets were downloaded from the Gene Expression Omnibus database (GEO, http://www.ncbi.nlm.nih.gov/geo). The following criteria were applied to select microarray datasets: expression profiles studies, the sample size of more than 5 samples, and human idiopathic pulmonary fibrosis tissues. Studies using nonhuman samples and a combination of expression profiles were excluded from the analysis. Gene expression datasets were retrieved using the key search terms “Idiopathic pulmonary fibrosis” from the GEO Datasets database. The datasets downloaded were GSE2052, GSE24206, GSE32537, and GSE110147. GSE2052 included 11 normal histology lung tissues and 14 idiopathic pulmonary fibrosis tissues. GSE24206 consisted of 6 normal histology lung tissues and 17 idiopathic pulmonary fibrosis tissues. GSE32537 included 50 normal histology lung tissues and 120 idiopathic pulmonary fibrosis tissues. GSE110147included 11 normal histology lung tissues and 22 idiopathic pulmonary fibrosis tissues. The following data were retrieved by two researchers working independently: type of reference, type of sample, gene expression data, number of cases and controls, platform, and GEO accession number. Any discrepancy in the data obtained between the two researchers was resolved by the involvement of a third researcher or through discussion. The study was approved by the Review Boards of the Binzhou Medical University.

### 2.2. Consensus Clustering

The “sva” package in R software (version 3.4) was employed to normalize raw data from the 4 datasets. Batch effects were removed from merged data as previously reported, followed by classification of the datasets into 2 categories: normal histology lung and IPF groups. Data quality control for batch effect was performed using principal component analysis. Consensus clustering was used to classify the IPF patients into different subgroups using the K means algorithm with the Spearman distance. The maximum cluster number was set as 10. The final cluster number was determined by the consensus matrix and the cluster consensus score (>0.8).

### 2.3. Comparison of the Clinical Characteristics between the Two Subgroups

Data on the gender, age, smoking history, and SGRQ score of IPF cases from the GSE32537 dataset were used to compare the clinical characteristics of the two subgroups. The chi-square test was used to analyze the data on gender and smoking history, while the unpaired *t*-test was used to analyze the data on age and SGRQ score.

### 2.4. Subgroup-Specific Differentially Expressed Genes

We then compared the gene expression patterns among the different subgroups to identify subgroup regulatory genes. The *T*-test was implemented with the R Limma package to determine differentially expressed genes. The cutoff selection criteria of the DEGs were *P* < 0.05 along with |log2fold change (FC)|>2.

### 2.5. Weighted Gene Co-Expression Network Analysis

The WGCNA method is commonly utilized to construct a scale-free weighted gene co-expression network based on subgroup-specific gene expression data. In this study, we used the R package “WGCNA” to identify potential functional modules that could characterize the biological function of each subgroup. In brief, we converted the adjacency matrix into a topological overlap matrix (TOM). The genes were then grouped into various modules using the TOM-based dissimilarity measure. The soft threshold for the scale-free network was determined based on the maximal R2 (power = 16). In the end, five functional modules were identified.

### 2.6. GO and KEGG Pathway Enrichment Analyses of Co-Expression Network Analysis Modules

Clusterprofiler was employed to determine the biological processes involved in the pathogenesis of IPF by identifying enriched functions and cascades. Clusterprofiler analyses and visualizes critical data on GO along with KEGG analyses. A *P* value <0.05 was considered to be significant.

### 2.7. Determination of Immune Infiltration with CIBERSORT

The CIBERSORT deconvolution algorithm was used to compare the infiltration levels of 22 immune cells among the molecular subgroups. Gene expression data were converted into levels of immune cells. Samples that showed *P* < 0.05  were selected. Correlation of immune cells in different subgroups was presented via a corheatmap plot. A violin plot was used to display the differences in expression of 22 infiltrating immune cells with the vioplot package of R. In the same subgroup, the correlation between different immune cells was analyzed by Spearman correlation analysis. For the same immune cells, the expression differences in different subgroups were analyzed by the unpaired *T*-test.

## 3. Results

### 3.1. Identification of Molecular Subgroups in Patients with IPF

**Figure 1:**
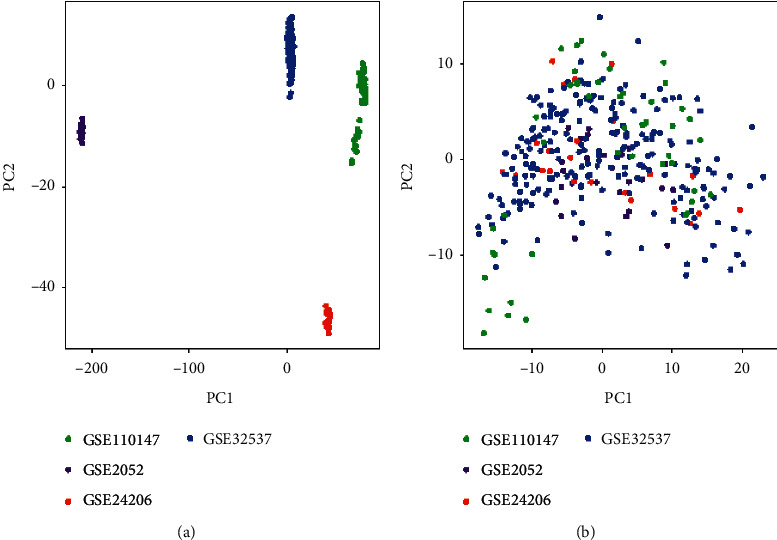
Principal component analysis of the 4 gene expression datasets. (a) Data before normalization. (b) Data following normalization.

**Figure 2:**
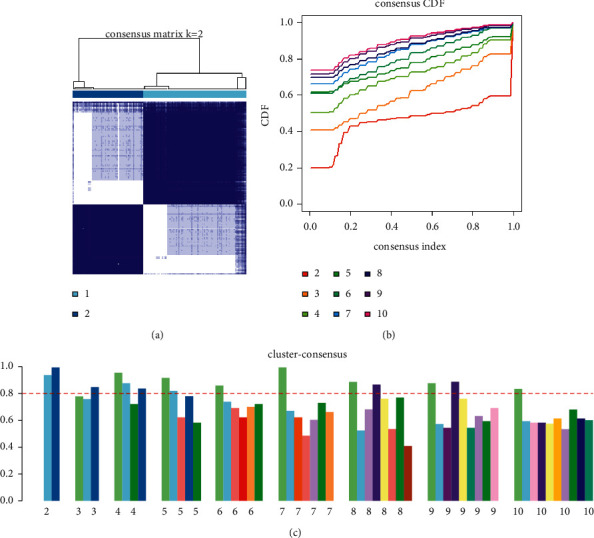
Consensus-based cluster analysis of gene expression in idiopathic pulmonary fibrosis. (a) The consensus matrix using a two-group model (*k* = 2). (b) The plot of the cumulative distribution function (CDF) for each number of clusters tested. (c) The consensus scores analysis of each cluster.

The Limma package was used to analyze the 4 IPF gene expression microarray datasets. Data preprocessing included background correction, normalization, and summarization. The scatter-plot based on PCA of normalized expression revealed that the batch effects arising from the use of different platforms had been removed (Figures 1(a) and 1(b)). Based on the gene expression data of 173 IPF patients, a consensus clustering algorithm was used to divide all samples into C1 and C2, with 103 and 70 samples, respectively (Figure 2(a)). The samples are divided into two categories based on the cumulative distribution function (CDF) and consistency score. CDF analysis showed that in the two categories, there was no significant increase in the area under the CDF curve (Figure 2(b)). At the same time, only in the two subgroups, the cluster consistency score of each subgroup was higher than 0.8, which shows that the classification with two subgroups is more robust than with the other subgroups (Figure 2(c)). Finally, two categories were selected for downstream analysis.

### 3.2. Comparing the Clinical Characteristics of Two Subgroups

**Figure 3:**
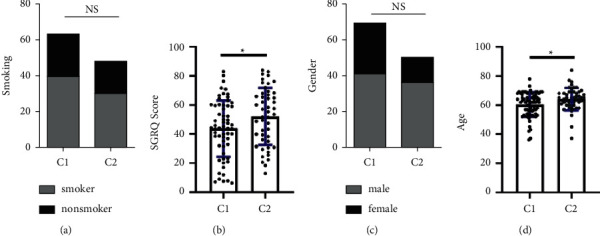
Comparison of clinical characteristics in different subgroups. (a, c) The status of gender and smoking in different subgroups is shown. (b, d) The status of age and SGRQ score in subgroups showed significant differences.

To further explore the clinical significance of the sample classification, we compared the age, gender, SGRQ score, and smoking history between the two groups of samples. We did not observe any significant differences in age and smoking history between the two groups (Figures 3(a) and 3(c)). However, the mean age (*P*=0.0147, Figure 3(d)) and the SGRQ score (*P*=0.0283, Figure 3(b)) were significantly higher in the C2 group than in the C1 group. Higher SGRQ scores indicate worse health status of the patient, suggesting that patients in the C2 group had a higher grade of IPF.

### 3.3. Molecular Characterization of the Molecular Subgroups

To identify the molecular differences between IPF subgroups, WGCNA was performed based on the expression levels of specifically upregulated genes in each subgroup. Differential expression analysis between the two subgroups identified 505 and 270 genes specifically regulated in subgroups C1 and C2, respectively (adjusted *P* < 0.05 and the absolute difference of means > 0.2).

**Figure 4:**
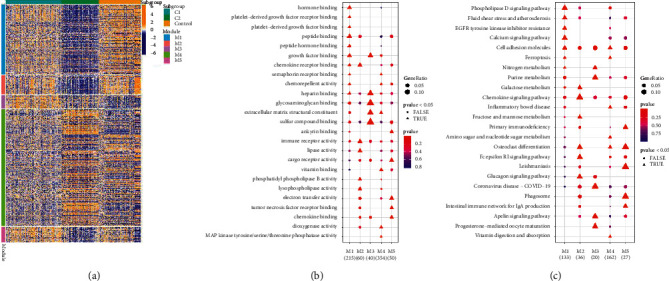
Molecular characterization of the molecular subgroups. (a) Expression heat map of five weighted gene co-expression network analysis modules. (b) GO analysis of 5 modules. (c) KEGG analysis of 5 modules.

The Limma package was used to further explore the molecular differences between the two groups. A total of 1751 differential genes were identified and classified into five modules, named M1–M5, using unsupervised clustering (Figure 4(a)). Gene set enrichment analysis revealed that the M4 gene module was mainly enriched in MAPK signaling and was mainly expressed in C2. The M1, M2, and M3 gene modules were mainly enriched in metabolic pathways and Chemokine signaling, while M5 was mainly enriched in phagosome inflammation. M1, M2, M3, and M5 were mainly expressed in C1 (Figures 4(b) and 4(c)).

### 3.4. Immune Cell Infiltration Landscape in the Molecular Subgroups

**Figure 5:**
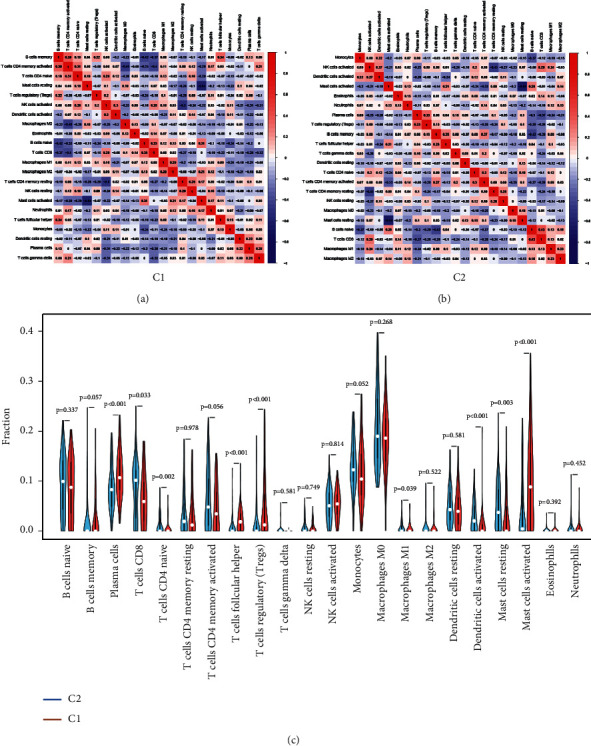
The difference of 22 immune cell infiltration proportions between C1 and C2. (a) The correlation matrix for 22 immune cell proportions in C1. (b) The correlation matrix for 22 immune cell proportions in C2. Red means positive correlation, blue means negative correlation, and the darker the color, the stronger the correlation. (c) Distribution of immune cells between C1 and C2. *P* values show the significance of distribution.

Analysis with the CIBERSORT algorithm revealed significant differences in the proportions of 22 infiltrating immune cell types between C1 and C2. C1 and C2 tissues were then filtered and used for further analysis, where 95 C1 and 66 C2 samples were left. The differences in immune cell proportions may be an intrinsic feature that can be utilized to characterize different differences. To further examine the overall expression pattern of infiltrating immune cells in C1 and C2 samples, correlation analysis between immune cells was undertaken. The correlation coefficient between 22 immune cells in C2 was observed, in which CD8 T cells and naive B cells had the strongest positive correlation (*r* = 0.43), whereas resting mast cells had the strongest negative correlation with activated mast cells (*r* = -0.64) (Figure 5(a)). However, these results were different from results obtained using C1 (Figure 5(b)). This outcome further proved the abovementioned speculation. This suggests a specific communication mode between immune cells. The violin plot illustrated the probable distribution of different immune cells in C1 and C2 tissues. Compared with C1 samples, C2 samples contained a higher proportion of M1 macrophages, resting mast cells, and CD8 T cells, whereas the proportions of activated mast cells, regulatory T cells (Tregs), and Plasma cells were relatively lower (Figure 5(c)). These results showed that the proportion of infiltrating immune cells might help to distinguish C1 from C2 subgroups.

## 4. Discussion

Idiopathic pulmonary fibrosis is a chronic and progressive fibrotic interstitial lung disease. The clinical course of IPF varies considerably and can be divided into three subtypes: slow progress, moderate progress, and rapid progress. However, the lack of a standard staging system to distinguish these subtypes makes it difficult to make clinical decisions such as when to initiate treatment, palliative care, and lung transplantation. Therefore, there is a need to define the molecular subtypes of IPF to support research on the pathogenesis of IPF. The aim is to stratify patients into groups with coherent and homogeneous genetic and molecular biomarker profiles. We used bioinformatics methods to analyze gene expression data of 173 IPF patients and explore the differences in molecular characteristics, age, gender, degree of inflammation, and related pathways among the subgroups of IPF patients. The goal was to identify the molecular subtypes of the disease that will help in the development of targeted therapy.

In this study, we successfully divided 173 IPF cases into two molecular subgroups based on gene expression profiles and found a significant correlation between clinical features and molecular subtypes. C2 cases had a higher SGRQ score and mean/median age than C1 cases. The Saint George Respiratory Questionnaire (SGRQ) is currently one of the most widely used scales for assessing the level of health impairment and quality of life of adult patients with respiratory diseases [8, 9]. The SGRQ questionnaire mainly measures the impact of chronic airflow limitations on the quality of life from the three dimensions of symptoms, activity capacity, and daily life impact [8]. The more severe the impact on life, the higher the weight and the greater the score. The high SGTQ score associated with C2 suggested that the cases in this group had more advanced IPF.

The role of molecular pathways in the pathogenesis, progression, and diagnosis of IPF is constantly being explored. The current theory believes that the pathogenesis of IPF is heterogeneous and involves epithelial injury, wound healing, innate and adaptive immunity, and inflammatory infiltration. In these macrocategories, many molecules interact in different pathogenic pathways and may become targets for new therapeutic drugs. It is worth noting that we identified 1751 differentially expressed genes in IPF patients, which were further classified into 5 modules, named M1–M5, using unsupervised clustering. Further analysis revealed that the modules were associated with a number of key pathways that regulate inflammation and metabolism. For example, M4 was enriched by MAPK signaling and B cell receptor signaling pathways. The MAPK signaling pathway is closely connected with IPF. Nie et al. [10] found that Shikonin suppresses the activation of pulmonary fibroblasts by regulating MAPK signaling pathways. TRB3 regulates mouse pulmonary interstitial fibrosis through the MAPK signaling pathway [11]. The M1 gene module was enriched by Phospholipase D signaling pathways. PLD has six subtypes, PLD1-6, with PLD1 and PLD2 subtypes having the ability to hydrolyze phospholipids. These two subtypes have been associated with various human pathophysiological processes, including cancer, hypertension, neurological diseases, diabetes, and acute lung injury [12–14]. Bleomycin can activate PLD in lung endothelial cells and lead to the production of reactive oxygen species, thereby regulating the process of lung fibrosis [15, 16]. The M5 gene module was mainly enriched in immune-inflammatory pathways, such as phagosome and PD-L1 expression. Wang et al. [17] found that PD1 affects the progression of pulmonary fibrosis by regulating Tregs. The effect of Gancao Ganjiang decoction is mediated in idiopathic pulmonary fibrosis by the PD-1 pathway [18].

CIBERSORT-based analysis of subtypes of infiltrating immune cells revealed marked differences between C1 and C2. There was a significant difference in the number of M1 macrophage cells, CD8 T cells, regulatory T cells (Tregs), and Plasma cells between C1 and C2. The CD8+ T cell is a type of cytotoxic T lymphocyte that secretes various cytokines to participate in immune function. CD8+ T cells have been associated with the levels of dyspnea and disease severity in IPF patients, suggesting that they may play a role in its pathogenesis [19–21]. Regulatory T cells (Tregs) are crucial in maintaining immune tolerance and immune homeostasis. The proportion of activated regulatory T cells is negatively correlated with severity of idiopathic pulmonary fibrosis [22]. Animal experiments also proved that regulatory T cells limit the deterioration of fibrosis in mice [23]. Over the past decade, macrophages have been shown to play a significant role in IPF pathogenesis. Depending on the local microenvironments, macrophages can be polarized to either classically activated (M1) or alternatively activated (M2) phenotypes [24]. Scholars have now applied nanoengineered immunosuppressive therapeutics to adjust the M1/M2 balance to enhance the treatment of idiopathic pulmonary fibrosis [25].

Although our study provided new insights into IPF, there were some limitations. First, our findings need to be interpreted with caution since they were not validated using in vitro or in vivo experiments. Second, since used publicly available data, we cannot guarantee the quality of the data.

## 5. Conclusions

Different IPF subgroups have unique gene expression patterns, which provide further insights into the pathogenesis of IPF. The expression patterns may also have predictive value.

## Data Availability

The underlying data supporting the results of our study can be found in the NCBI platform and downloaded from the Gene Expression Omnibus database (GEO, http://www.ncbi.nlm.nih.gov/geo).
